# Case Report: Hepatitis B virus reactivation following rituximab therapy in a patient receiving maintenance hemodialysis

**DOI:** 10.3389/fmed.2026.1808087

**Published:** 2026-06-30

**Authors:** Ruocheng Luo, Fu Qiao, Huaihong Yuan, Peixuan Huang, Yi Chen, Shiyu Li

**Affiliations:** 1 Center of Infectious Diseases, West China Hospital, Sichuan University, Chengdu, China; 2 Department of Infection Control, West China Hospital, Sichuan University, Chengdu, China; 3 Department of Nephrology, West China Hospital, Sichuan University, Chengdu, China

**Keywords:** hepatitis B virus reactivation, hospital infection management, infection control, maintenance hemodialysis, prevention strategies, risk assessment

## Abstract

**Objective:**

To investigate a case of hepatitis B virus reactivation (HBV-R) in a post-lymphoma patient with renal transplantation and long-term maintenance hemodialysis (MHD), and to explore prevention strategies for high-risk populations.

**Methods:**

Patient records were reviewed for dialysis history, HBV markers, and liver function. Environmental sampling was conducted, and a multidisciplinary team established individualized management as well as optimized screening and vaccination strategies.

**Results:**

The patient had undergone maintenance hemodialysis since February 2023 with stable hepatitis B virus (HBV) serology. In March 2024, hepatitis B surface antigen (HBsAg), hepatitis B e antigen (HBeAg) and HBV DNA tested positive. The patient had received intermittent chemotherapy and rituximab at another hospital during dialysis. Following reactivation, the patient was transferred to dedicated dialysis equipment with isolation nursing care, strict infection control measures, and environmental monitoring. A multidisciplinary team refined high-risk patient screening and reinforced vaccination programs.

**Conclusion:**

Hemodialysis patients under prolonged immunosuppression face elevated HBV reactivation risk. Early identification and risk-based management are essential to reduce adverse impacts on patient safety and healthcare quality.

## 1 Background

The widespread clinical utilization of biologic agents has led to the extensive application of rituximab, an anti-CD20 monoclonal antibody, in the treatment of hematologic malignancies, autoimmune diseases, and certain kidney disorders due to its potent immunosuppressive properties. However, its strong B-cell depleting activity also increases the risk of hepatitis B virus reactivation (HBV-R) (1–3). Patients undergoing maintenance hemodialysis (MHD) represent a high-risk population for HBV infection due to immunodeficiency, frequent exposure to blood and blood products, use of shared dialysis equipment, and inadequate implementation of infection control measures (4–6). The use of rituximab in this patient group further compromises immune control of HBV, thereby heightening the risk of HBV-R and the potential for outbreaks among hemodialysis patients.

Previous studies have indicated that rituximab is associated with a HBV-R rate ranging from 6.3 to 27% in patients with hematologic malignancies (7–9) Among patients with rheumatoid arthritis, the reactivation rate was found to be 9.1% in those who were HBsAg-negative but anti-HBc-positive (10, 11). Furthermore, even a single administration of rituximab may trigger HBV-R in renal transplant recipients (12, 13). However, data concerning patients on maintenance hemodialysis remain limited. In this study, we present and analyze a case of HBV-R induced by rituximab in a patient with a history of renal transplantation, brain tumor, multiple comorbidities, and long-term maintenance hemodialysis. By integrating the latest domestic and international guidelines along with relevant literature, this report aims to identify high-risk populations for HBV-R among hemodialysis patients and to explore strategies for optimizing infection control and management to mitigate the risk of HBV transmission.

The patient was a 55-year-old male with a history of renal transplantation performed in 2019 due to chronic renal failure. Postoperatively, tacrolimus was initiated in July 2019 as maintenance immunosuppressive therapy. In December 2022, during the perioperative period of intracranial lymphoma resection, immunosuppressive therapy consisted of a combination of tacrolimus and sirolimus. From January 2023 onward, tacrolimus was discontinued and sirolimus was maintained as monotherapy. The patient continued sirolimus during the period of renal graft dysfunction and after initiation of maintenance hemodialysis.

This patient had a 20-year history of hypertensive nephropathy, which was adequately managed with bisoprolol, terazosin, and nifedipine. Additionally, he was diagnosed with type 2 diabetes mellitus 8 months prior and was treated with empagliflozin with satisfactory glycemic control. The patient had no history of tuberculosis or other infectious diseases, nor any known food or drug allergies. In October 2022, the patient underwent neurosurgical resection for an intracranial lymphoma at West China Hospital, followed by two cycles of postoperative chemotherapy. In late February 2023, the patient was admitted for pulmonary infection and progressive graft dysfunction, and maintenance hemodialysis was initiated thereafter.

**Figure 1:**
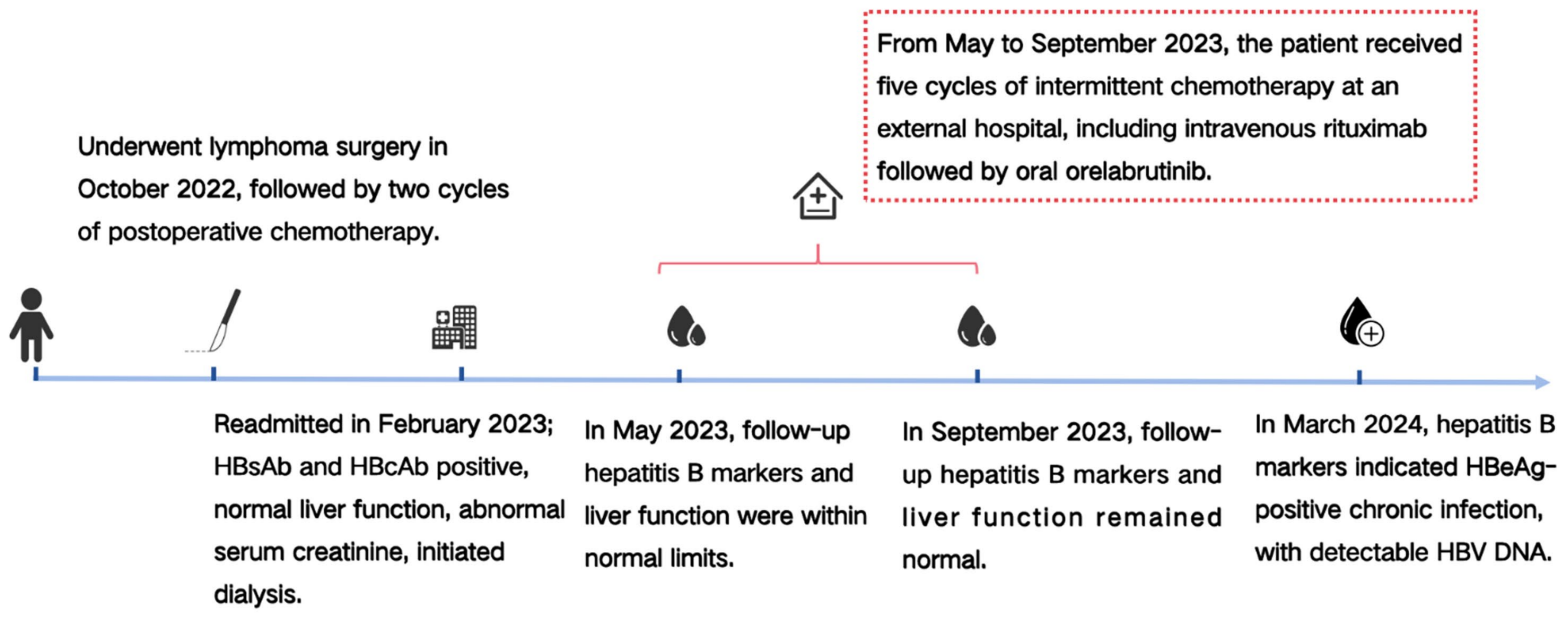
Schematic overview of patient diagnosis and treatment.

Screening for HBV serological markers at admission revealed positivity for hepatitis B surface antibody (HBsAb) and hepatitis B core antibody (HBcAb), while all other markers were negative. Biochemical tests showed alanine aminotransferase (ALT) of 7 IU/L, aspartate aminotransferase (AST) of 27 IU/L, with no significant abnormalities in alkaline phosphatase (ALP) or *γ*-glutamyl transferase (GGT), although serum creatinine was elevated to 711 μmol/L. After discharge, the patient continued to receive hemodialysis at our hospital once per week. According to the Standard Operating Procedures for Blood Purification (2021 edition, National Health Commission of China) (14), newly initiated hemodialysis patients are required to undergo immediate screening for infectious disease markers and retesting within 3 months. The patient remained on maintenance hemodialysis at our center, and in May, repeat HBV serological testing showed positivity for HBsAb and HBcAb with all other markers negative, consistent with a history of prior HBV infection. Liver function tests remained within the normal limits. In June 2023, liver function tests revealed elevated ALT at 102 IU/L, AST at 48 IU/L, ALP at 136 IU/L, and GGT at 77 IU/L, with an AST/ALT ratio of 0.47, indicating hepatocellular injury. No dynamic monitoring of HBV serological markers was performed during this dialysis period. In September 2023, the patient underwent routine retesting of infectious disease markers, which showed no change from previous results, and liver function tests were within normal limits. In March 2024, repeat HBV serological testing indicated HBsAg at 52.5 COI, HBeAg at 23.0 COI, and HBcAb at 0.519 COI, while all other serological markers were negative. The HBV DNA quantification was 1.16 × 10^4^ IU/mL, indicating HBV seroconversion. A field investigation revealed that between May and September 2023, during maintenance hemodialysis at our hospital, the patient received 5 cycles of intermittent chemotherapy and intravenous rituximab at an outside hospital, later transitioning to oral orelabrutinib. The treatment timeline is illustrated in Figure 1.

## 2 Methods

### 2.1 Epidemiological investigation

Following the notification from the Laboratory Medicine Department, the Department of Infection Control immediately initiated an epidemiological investigation within the department of hemodialysis unit. The task was carried out by the hospital’s Emergency Epidemiological Investigation Team, which has been established since 2020. The investigation was conducted simultaneously in three areas.

First, a comprehensive review of the patient’s medical records was conduct and an on-site interview was perform by both infection control practitioner and the patient’s attending physician to identify potential risk factors for infection. Second, the patient’s dialysis records over the past 6 months were analyzed to identify other patients who used the same dialysis machine, followed by hepatitis B marker testing for these individuals. Third, environmental surveillance culture for hepatitis B virus contamination was perform, encompassing the surfaces of hemodialysis equipment, headboards, footboards, bed rails, bed crank handles, bedsheets, and mattresses used by the patient, as well as those of neighboring and opposite-bed patients (15). Using the swab method (16), all the samples were sent the Department of Laboratory Medicine and tested through real-time fluorescence polymerase chain reaction (qPCR).

### 2.2 Infection prevention and control measures

#### 2.2.1 Convene an emergency multi-department coordination meeting to discuss prevention and control measures

An Emergency Multi-Department Coordination Meeting was convened by the chairperson of the hospital’s Infection Control Committee. Infection control personnel, nephrology specialists, dialysis experts, head dialysis nurses, infectious disease treatment experts, medical laboratory specialists, and medical administrators were invited to discuss emergency response measures and establish protocols for the early identification of at-risk patients and the implementation of effective control measures.

#### 2.2.2 Patient placement and hepatitis B marker monitoring

Due to the absence of a designated hepatitis-positive dialysis area within the patient’s current hospital campus, he was transferred to a single room with a reserved dialysis machine and receive care from a dedicated special care team for his next dialysis session. After confirming the patient’s HBV was positive, he was transferred to the hepatitis B-positive dialysis area at another campus to continue dialysis. According to the procedure, the patient’s HBV markers was tested using the ELISA method every 6 months to assess whether seroconversion has occurred.

#### 2.2.3 Cleaning and disinfection

The hemodialysis machine used by the patient was immediately disinfected internally using citric acid for pipeline cleaning (17). Simultaneously, all the surface of the hemodialysis machine and the patient beds were cleaned by nurses using towels containing 2000 mg/L chlorine disinfectant (18, 19).

#### 2.2.4 Long-term multidisciplinary strategy for managing HBV reactivation risk in high-risk populations

Given that patients with chronic hepatitis B undergoing MHD and receiving antineoplastic agents or immunosuppressive therapy constitute a high-risk population for HBV-R, targeted strategies are essential for early detection and precise prevention. Accordingly, a series of multidisciplinary expert consultations—engaging specialists from the Infectious Diseases Center, Department of Laboratory Medicine, Department of Nephrology, and Hemodialysis Unit—was convened to establish a sustainable, hospital-wide mechanism for HBV-R risk screening and management in this population, thereby ensuring timely and effective implementation of infection control measures.

## 3 Results

### 3.1 Antiviral therapy and health monitoring of the patient

**Figure 2:**
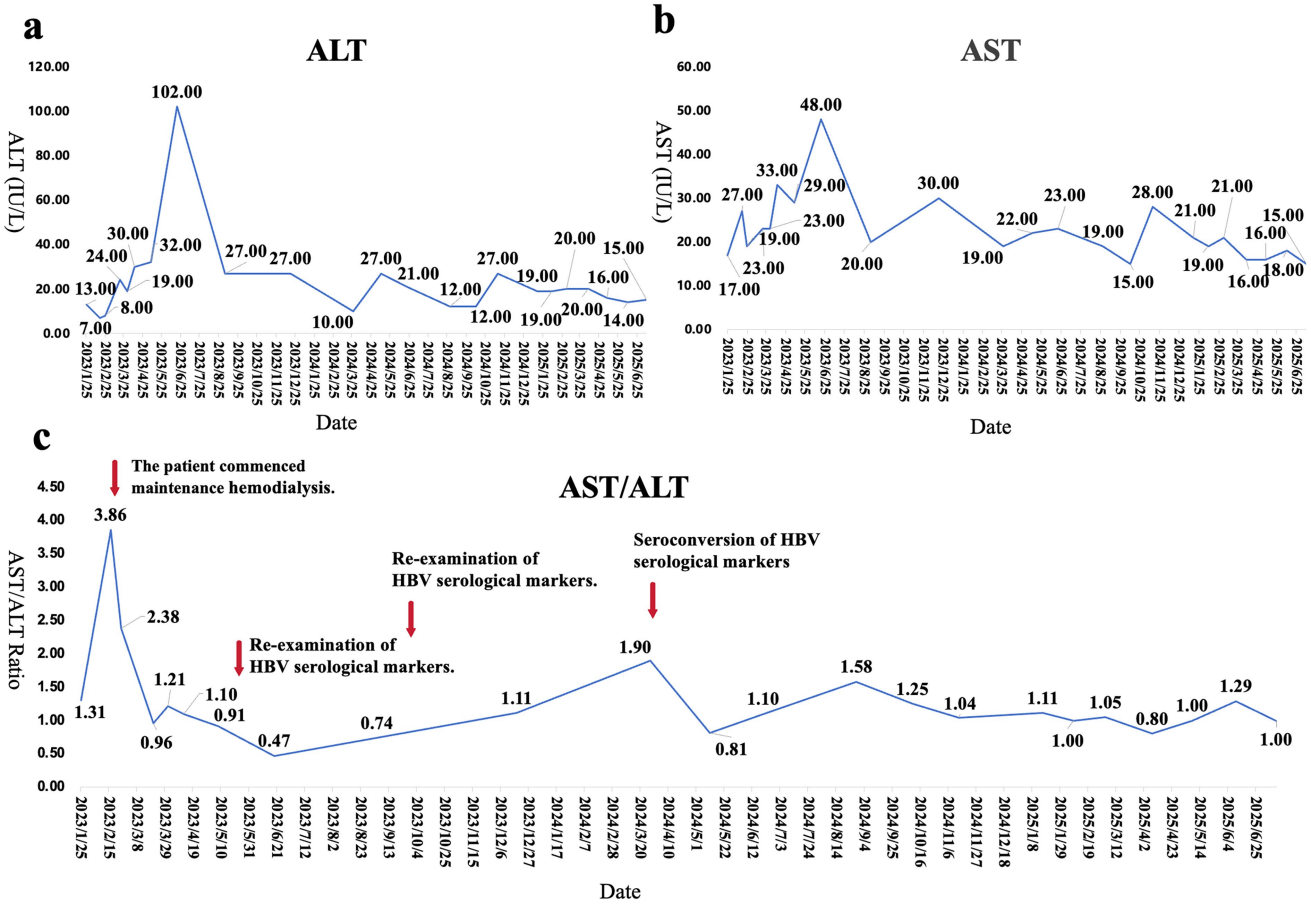
Trends in liver function tests and HBV DNA levels during the hemodialysis treatment. **(a)** Changes in ALT. **(b)** Changes in AST. **(c)** Changes in the AST/ALT ratio.

Following the reversion of HBV serological markers, a combination antiviral therapy consisting of tenofovir alafenamide fumarate and entecavir was initiated. Throughout the treatment, HBV DNA levels and liver function tests were monitored regularly, revealing no significant drug-related adverse events observed, and the therapy was well tolerated. By July 2024, follow-up testing indicated undetectable HBV DNA, and the patient has since continued maintenance hemodialysis at our hospital. Liver function tests conducted during hemodialysis, along with HBV DNA monitoring results, are summarized in Figure 2.

### 3.2 Screening of linked patient

The epidemiological investigation identified 79 patients who had used the same dialysis machine as the index case within the preceding 6 months. All patients underwent HBV serological testing in batches within one week, which included HBsAg, HBsAb, HBeAg, HBeAb, and HBcAb, revealing six distinct serological profiles; however, all patients tested negative for HBsAg (Table 1). Follow-up testing of the same cohort at 3 and 6 months demonstrated that all patients remained HBsAg-negative, with no changes observed in other serological profiles from the initial results.

**Table 1:** Screening results of HBV serological markers in associated patients.

Serial number	HBsAg	HBsAb	HBeAg	HBeAb	HBcAb	Count	Propotion (%)
1	−	+	−	−	+	20	25.32
2	−	−	−	−	−	18	22.78
3	−	+	−	−	−	16	20.25
4	−	+	−	+	+	14	17.72
5	−	−	−	−	+	9	11.39
6	−	−	−	+	+	2	2.53
Total						79	100.00

### 3.3 Environmental sampling and analysis

A total of 30 environmental specimens were collected, including four samples each from the headboard, footboard, bed rails, bed crank handle, bed sheet, mattress, and hemodialysis machine surface, as well as two samples from the equipment belt. HBV nucleic acid testing of all specimens yielded negative results.

### 3.4 Optimization of risk screening strategies for high-risk Hemodialysis patients

The hospital revised the “Infection Prevention and Control Policy for the Hemodialysis Unit” to specify the screening content and principles for newly admitted or transferred hemodialysis patients. To facilitate the timely identification of high-risk individuals, an admission reporting system was established to collect patients’ prior infectious disease marker results, history of antineoplastic or immunosuppressive therapy, and other relevant medical history, as detailed in Box 1.

Box 1 **Risk screening strategy for high-risk hemodialysis patients.**

**Table:** 

Screening population	Screening content	Screening frequency/Measures
Newly initiated or newly transferred hemodialysis patients	HBV serological markers; for HBsAg-negative and HBcAb-positive patients, perform high-sensitivity HBV-DNA testing	At admission
HBcAb-positive patients receiving chemotherapy or immunosuppressive therapy (within ≤18 months after drug withdrawal, without prophylactic anti-HBV treatment)	HBV serological markers	Monthly
Chronic hepatitis B liver transplant recipients (HBsAg-negative, HBcAb-positive)	HBV serological markers	Monthly
HBsAb-negative patients	HBV serological markers	Every 3 months
Patients with isolated HBcAb positivity	HBV serological markers	Every 3 months
Patients with unexplained abnormal liver function	HBV serological markers; for HBsAg-negative and HBcAb-positive patients, perform high-sensitivity HBV-DNA testing	Test upon ALT elevation; if HBV-DNA positive, dynamically monitor viral load
Patients negative for all HBV serological markers and without vaccination contraindications	HBV vaccination history	HBV vaccination and follow-up of immune response; those refusing vaccination must sign informed consent

## 4 Discussion

MHD is the primary therapeutic modality for patients with end-stage renal disease (ESRD). By diverting blood outside the body, it enables solute exchange through diffusion or convection, thereby removing metabolic waste products, maintaining electrolyte and acid–base balance, and sustaining life (20). However, patients with ESRD have severely impaired renal function, compromised immunity, prolonged extracorporeal circulation, and require frequent vascular access punctures (21), all of which place them at an exceptionally high risk of infection (22). Among MHD patients with a history of HBV infection, the concomitant use of high-risk antineoplastic agents, such as targeted therapies or immunosuppressants, during dialysis can precipitate HBV-R. Evidence indicates that in patients with chronic HBV infection but inactive viral replication, HBV carriers or those in the convalescent phase of HBV, immunosuppressive therapy may suppress immune responses and subsequently trigger HBV reactivation (23).

Rituximab, a chimeric human–mouse monoclonal antibody that target CD20, is a first-line immunosuppressive agent for the treatment of non-Hodgkin’s lymphoma (24). By binding to CD20 on B lymphocytes, rituximab induces B-cell lysis and impairs their antigen-presenting function, thereby exerting immunosuppressive effects (25). However, B lymphocytes play a critical role in mounting antiviral responses against HBV, as they produce various HBV-specific antibodies and serve as essential intermediaries in CD4 + T lymphocyte–mediated antiviral activity (26). Host treatment history, viral replication status, and immune competence are key determinants of HBV-R (27). Evidence indicates that HBV-R frequently occurs during immunosuppressive therapy for hematological malignancies and various solid tumors, with clinical manifestations ranging from mild transaminase elevation to fulminant hepatic failure. Notably, HBV-R is particularly common among patients receiving rituximab-based antineoplastic therapy (28).

The use of immunosuppressive agents compromises the host immune system, impairing its ability to control latent HBV and thereby leading to HBV-R. HBV-R not only exacerbates hepatic injury but may also increase the risk of blood-borne transmission of infectious diseases during the hemodialysis process. According to the Procedure (14), long-term dialysis patients are required to undergo screening for infectious disease markers every 6 months. However, this routine screening frequency is insufficient to adequately cover populations at high risk for HBV-R, resulting in delayed recognition and intervention, and representing a critical gap in the prevention and control of blood-borne infections. Therefore, for patients receiving long-term MHD in combination with immunosuppressive therapy, more targeted surveillance and preventive strategies are warranted.

The emergency management of this case prompted a comprehensive review of infection control practices in the hemodialysis unit. The incident revealed that current protocols insufficiently address high-risk subgroups, particularly patients with prior immunosuppressive exposure, coexisting malignancies, negative HBsAb or positive HBcAb serology, and unexplained liver function abnormalities. These findings underscore the need to expand beyond existing national guidelines by incorporating risk-stratified surveillance, dynamic HBV marker monitoring, and reinforced vaccination management. Such supplementary measures are critical for the early identification of vulnerable populations and the timely implementation of preventive interve.

The delayed identification of rituximab exposure in this case was primarily attributable to chemotherapy and immunosuppressive treatment administered at an external healthcare institution without timely transfer of treatment records to the dialysis center. This highlights a gap in cross-institutional information sharing for high-risk therapies. Incorporating exposure to high-risk immunosuppressive agents, including rituximab and other biologic therapies, into routine dialysis medical records and dialysis-based electronic information systems, together with timely updates from external treatment facilities, may facilitate earlier risk stratification and implementation of preventive interventions before HBV serological conversion occurs (29, 30).

Importantly, vaccination remains a key component of HBV prevention in hemodialysis populations and should be integrated into infection control strategies. According to *Expert Recommendations on Hepatitis B Vaccination in Adults* (31), HBV-susceptible patients should receive an enhanced vaccination schedule (0, 1, 2, and 6 months, 20 μg per dose), followed by post-vaccination anti-HBs testing. Additional vaccine doses or booster vaccination should be administered in patients who fail to achieve or maintain protective antibody levels (31). If the anti-HBs level remains <10 mIU/mL, an additional three doses of 20 μg hepatitis B vaccine or one dose of 60 μg recombinant yeast-derived hepatitis B vaccine is recommended, with repeat anti-HBs testing performed 1–2 months after completion of the second vaccination series. For persistent non-responders, one additional dose of 60 μg recombinant yeast-derived hepatitis B vaccine may be considered (31).

In summary, this study established a systematic screening and management strategy for high-risk patients. For newly initiated or transferred hemodialysis patients, pre-admission infectious disease screening was reinforced and an admission reporting system was implemented to enable early identification of high-risk individuals. For patients with a history of immunosuppressive or other high-risk drug exposure, specific underlying diseases, HBsAb negativity or HBcAb positivity, or unexplained liver function abnormalities, the frequency of HBV marker monitoring was increased. In addition, HBV vaccination efforts and their management were strengthened to enhance population-level immune protection, thereby preventing nosocomial infections and safeguarding medical quality and patient safety.

As a single case report, this study cannot establish causality. The association between rituximab exposure and HBV reactivation was inferred from the clinical course and existing evidence. Further multicenter studies with larger sample sizes are warranted to better characterize the risk of HBV reactivation and validate the proposed prevention and management strategies.

## Data Availability

The original contributions presented in the study are included in the article/supplementary material, further inquiries can be directed to the corresponding author.
